# Glycosylation characterization of therapeutic mAbs by top- and middle-down mass spectrometry

**DOI:** 10.1016/j.dib.2015.11.031

**Published:** 2015-11-24

**Authors:** Bao Quoc Tran, Christopher Barton, Jinhua Feng, Aimee Sandjong, Sung Hwan Yoon, Shivangi Awasthi, Tao Liang, Mohd M. Khan, David P.A. Kilgour, David R. Goodlett, Young Ah Goo

**Affiliations:** a Department of Pharmaceutical Sciences, School of Pharmacy, University of Maryland, Baltimore, MD, USA; b MedImmune, LLC, Gaithersburg, MD, USA

## Abstract

A reference monoclonal antibody IgG1 and a fusion IgG protein were analyzed by top- and middle-down mass spectrometry with multiple fragmentation techniques including electron transfer dissociation (ETD) and matrix-assisted laser desorption ionization in-source decay (MALDI-ISD) to investigate heterogeneity of glycosylated protein species. Specifically, glycan structure, sites, relative abundance levels, and termini structural conformation were investigated by use of Fourier transform ion cyclotron resonance (FT-ICR) or high performance liquid chromatography electrospray ionization (HPLC-ESI) linked to an Orbitrap. Incorporating a limited enzymatic digestion by immunoglobulin G-degrading enzyme *Streptococcus pyogenes* (IdeS) with MALDI-ISD analysis extended sequence coverage of the internal region of the proteins without pre-fractionation. The data in this article is associated with the research article published in *Journal of Proteomics* (Tran et al., 2015) [1].

**Specifications Table**

**Table:** 

Subject area	Biology, Chemistry
More specific subject area	Top- and middle-down mass spectrometric analysis of protein species and proteoforms
Type of data	Table, Figure
How data was acquired	Mass spectrometry data were collected on a 12TFT-ICR XR (Bruker) and a LTQ-Orbitrap Elite (Thermo Scientific)
Data format	MS spectra
Experimental factors	Top- and middle-down analyses with multiple fragmentation techniques including electron transfer dissociation (ETD) and matrix-assisted laser desorption ionization in-source decay (MALDI-ISD) for characterization of a reference monoclonal antibody IgG1 and a fusion IgG protein
Experimental features	Glycan profiling including structure, sites, relative abundance levels, and termini structural conformation were investigated
Data source location	Baltimore and Gaithersburg, Maryland, USA
Data accessibility	Data is provided within this article

**Value of the data**

• Application of top-down and middle-down mass spectrometry for characterization of comprehensive glycosylated protein species from recombinant IgG and IgG-fusion proteins.
• Detection of glycan structure, sites, and relative abundance levels.
• Investigation of termini structural conformation; N-terminal glutamine (Q) to pyroglutamate (pyroGlu, aka. pE) conversion and C-terminal lysine truncation.
• Improvement on protein sequencing by MALDI ISD, ETD in combination with middle-down analysis by use of an enzyme, IdeS.

## 1 Data

Glycosylation profiling of recombinant IgG and IgG-fusion proteins and sequence coverage information.

## 2 **Experimental design, materials and methods**

The recombinant human mAb (IgG1) and IgG1-fusion protein were manufactured using standard cell culture, purification and formulation processes [2]. The IgG1 has two identical light chains and two identical heavy chains with molecular weight of 150 kDa and N-glycosylation site at N297. The IgG fusion protein, approximately 90 kDa, was comprised of two chains each with a human protein domain fused onto the N-terminus of a human IgG1 constant domain (Fc). The protein has three N-glycosylation sites at N76, N108 and N207.

## 3 Analysis of the IgG protein

The IgG protein was analyzed 1) in reduced form by MALDI-ISD in a 12 T Fourier Transform Ion Cyclotron Resonance (FT-ICR XR) mass spectrometer (Bruker, Bremen, Germany), and 2) after IdeS digestion and dithiothreitol (DTT) reduction to generate three smaller fragments; light chain, Fab, and Fc/2 after which analyzed by LC–MS/MS on an Orbitrap Elite (Thermo Scientific, Saint Jose, CA, USA).

Intact protein mass measurement for detection of protein species of the IgG was performed using ESI FT-ICR MS as demonstrated in the related study [1].

### 3.1 MALDI-ISD analysis of reduced protein

**Fig. 1:**
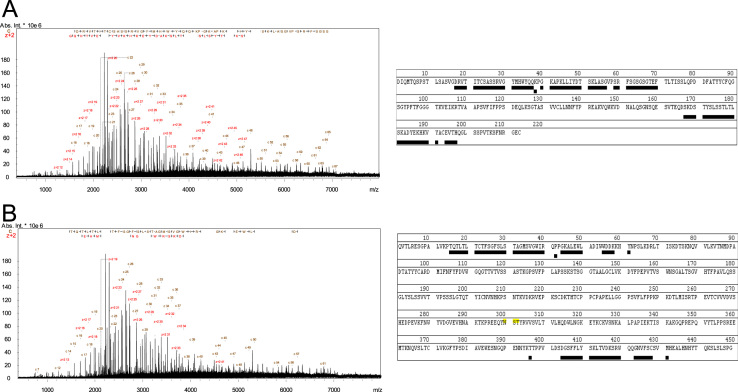
MALDI-ISD mass spectrum of the DTT-reduced IgG1 mAb. Amino acid residues underlined were identified after phase correction by c or z+2 ions with a mass error threshold of 50 ppm, covering (A) 37.6% of the light chain and (B) 14.3% of the heavy chain.

The IgG protein in 1 µg/µL in 50 mM ammonium bicarbonate buffer was reduced to light and heavy chain by 20 mM dithiothreitol (DTT, product #43815, Sigma-Aldrich, St. Louis, MO, USA) at 80 °C for 15 min. The reduced protein sample was buffer exchanged to 5% acetonitrile (ACN) /0.1% formic acid (FA) using a 10 kDa molecular weight cut off (MWCO) centrifugal filter (product # UFC201024, Merck Millipore, Tullagreen, Carrigtwohill, Ireland) and then diluted to 17 µg/µL in 50% ACN/0.1% FA. 1 µL sample and 1 µL of 1,5-diaminonaphthalene (1,5-DAN, product # 56451, Fluka, St. Louis, MO, USA) saturated in ACN were spotted on a stainless steel MALDI plate. MALDI-ISD experiment was performed with a transient domain of 1 mega-word data points (estimated resolving power of 270,000 at *m*/*z* 400) covering *m*/*z* range 400–10,000. MALDI-ISD mass spectra after phase correction [3] were processed to identify c- and z+2 ions from light chain and heavy chain with mass tolerance 50 ppm using DataAnalysis 4.2 and Biotools software 3.2 (Bruker) (Fig. 1).

### 3.2 LC-MS/MS analysis of IdeS-digested protein

**Fig. 2:**
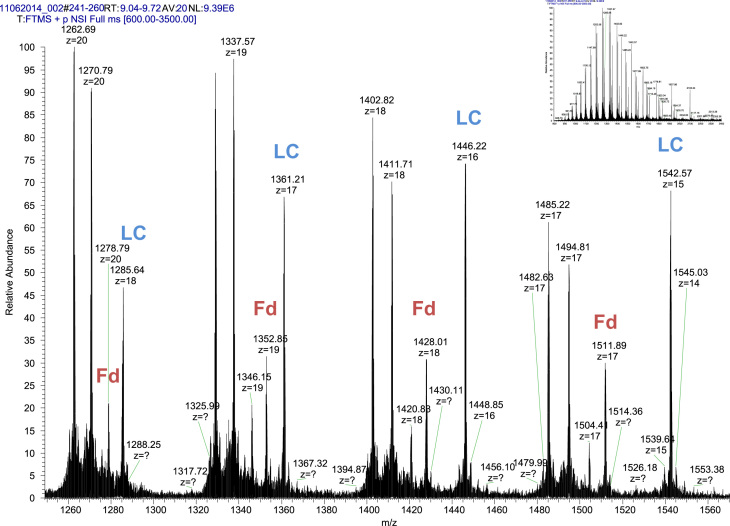
LC-MS/MS mass spectrum of IdeS/DDT treated IgG mAb. The charge state distribution of light chain (LC) and Fd domain are shown. Inset shows the full mass spectrum collected after accumulation of 20 scans over an *m*/*z* range 600–3500 at a resolving power of 120,000.

**Fig. 3:**
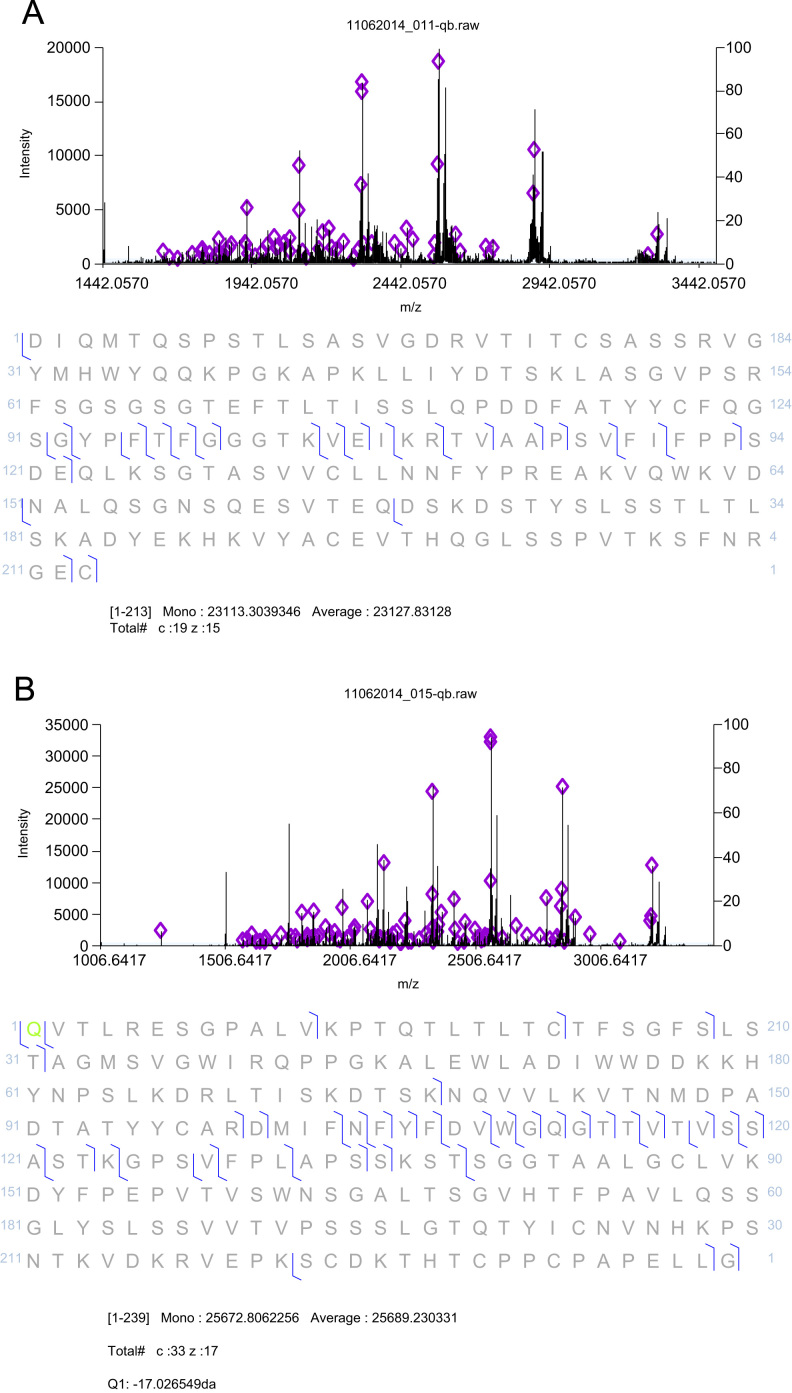
(A) ETD mass spectrum of precursor *m*/*z* 1446 (charge 16+) was searched against IgG1 mAb light chain sequence with mass tolerance of 100 ppm. ETD mass spectrum was produced from accumulation of 49 scans. Identified c- and z- fragment ion series are indicated in diamonds in the spectrum and relevant sequence identification is shown in the sequence. (B) ETD mass spectrum of precursor *m*/*z* 1512 (charge 17+) was searched against Fd sequence of IgG1 mAb with mass tolerance of 100 ppm. ETD mass spectrum was produced from accumulation of 50 scans. Identified c- and z- fragment ion series are indicated in diamonds in the spectrum and relevant sequence identification is shown in the sequence. N-terminal glutamine (Q) to pyroglutamate (pyroGlu) conversion was confirmed with precursor mass error 3.9 ppm (0.10 Da).

The IgG protein was digested with IdeS (product # A0-FR1-020, Genovis AB, Lund, Sweden) at a ratio of 1 unit of enzyme per 1 µg protein for 2 h at 37 °C, followed by DTT treatment. Approximately 14 µg of the IdeS/DTT treated sample was separated on a 100 µm×20 mm column packed with Jupiter 5 µm C5 particles (product # 04A-4052, Phenomenex, Torrance, CA, USA) by a fast mobile gradient of 5–50% ACN/0.1% FA in 15 min at a flow rate of 0.3 µL/min. Light chain and Fd domain were detected in a range of 350–3500 *m*/*z* at resolving power of 120,000 in positive ion mode on the Orbitrap Elite (Fig. 2). Precursor masses were selected for ETD fragmentation. ETD spectra were acquired with 5 microscans at resolving power of 120,000 and processed by MASH Suite version 1.0 software [4]. The fragment ions and corresponding sequence coverage are shown in Fig. 3A for the light chain and Fig. 3B for the Fd domain. Glutamine (Q) to pyroglutamate (pyroGlu) conversion was confirmed on the N-terminal of heavy chain (Fig. 3B).

## 4 Analysis of the IgG fusion protein

The fusion protein was analyzed 1) by accurate mass measured in FT-ICR for protein species detection 2) by MALDI-ISD following DTT reduction in FT-ICR, and 3) by accurate mass measurement following IdeS cleavage in FT-ICR or ETD MS/MS in the Orbitrap Elite.

### 4.1 Accurate mass measurement of intact protein

**Fig. 4:**
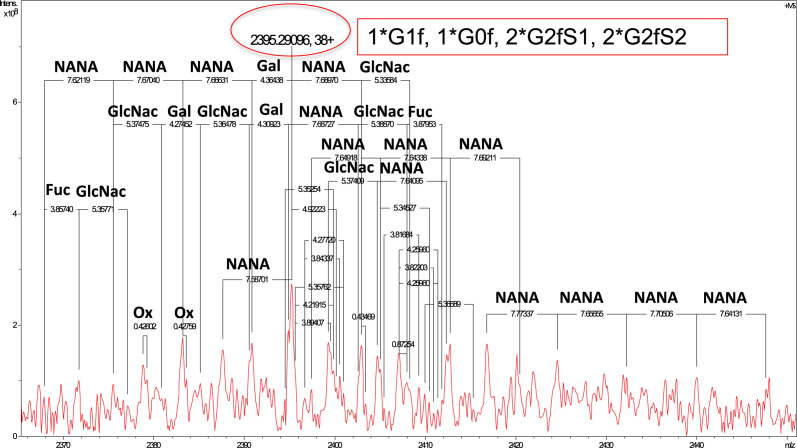
Fourier transform ion cyclotron resonance (FT-ICR) mass spectrum of the intact fusion protein peaks (charge 38+). The mass spectrum was produced from accumulation of 1650 scans collected in a time domain of 128 K words data points. Major peaks demonstrate a heterogeneous combination of glycosylation, predominantly by sialic acids (NANA). Additional information regarding the glycan nomenclature and structure are provided in Fig. 5.

**Fig. 5:**
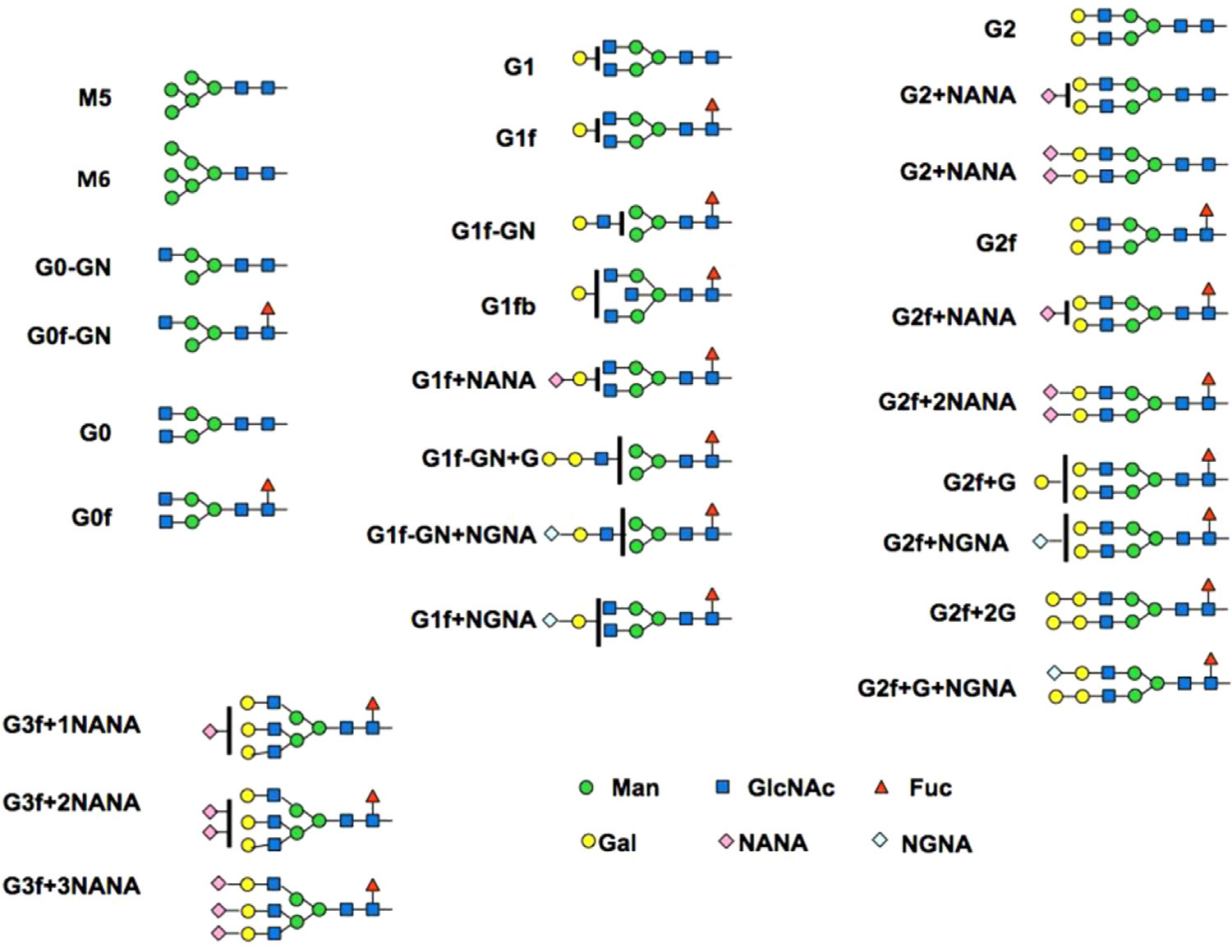
The glycan nomenclature and structure.

The fusion protein sample was buffer exchanged to 5%ACN and 0.1%TFA using Amicon 50 kDa MWCO centrifugal filters (product # UFC505024, Merck Millipore). Since the fusion protein sample was stored in buffer with detergent, detergent removal was performed by Pierce detergent removal kit (product # 87778, Thermo Scientific, Rockford, CA, USA). The detergent-free protein sample was diluted to 10 µg/µL final concentration in 50% ACN/0.1% FA and introduced to electrospray ionization (ESI)-FT-ICR for intact protein mass measurement for detection of protein species. The experiment was carried out in positive ion mode on the 12 T FT-ICR at flow rate 2 µL/min. Mass spectra were collected in magnitude mode with a transient domain of 128,000 word data points to cover *m*/*z* range 400–4000, with an accumulation of 1650 scans. Fig. 4 shows the protein species detected in *m*/*z* region 2365–2450 with high heterogeneity of glycosylation. Corresponding glycan nomenclature and structures are shown in Fig. 5.

### 4.2 MALDI-ISD analysis of reduced protein

**Fig. 6:**
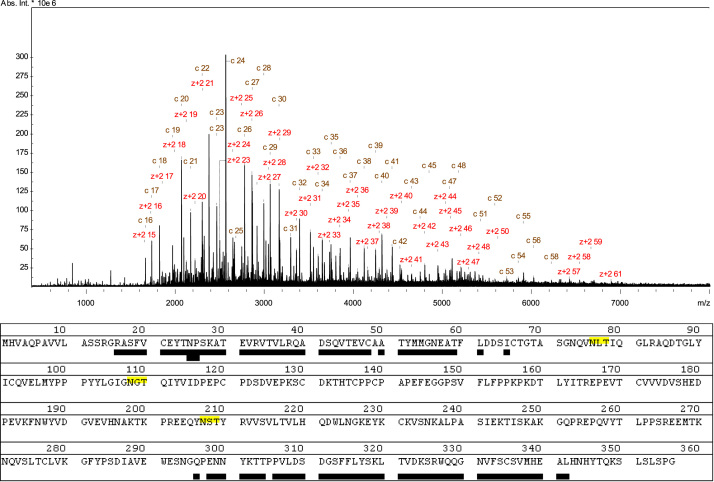
MALDI-ISD mass spectrum of the DTT reduced fusion protein. Underlined sequence indicates the amino acid residues identified by phase-corrected c and/or z+2 ions, which showed an overall sequence coverage of 25.3% of the full sequence.

The protein sample was diluted to 1 µg/µL in in 50 mM ammonium bicarbonate buffer and reduced with 20 mM DTT at 80 °C for 15 min. The reduced sample was then detergent removed. The detergent-free protein was diluted in 50% ACN/0.1% FA at a final concentration of 7 µg/µL. 1 µL of sample and 1 µL of DAN matrix were spotted on stainless steel MALDI plate. MALDI-ISD mass spectra were recorded for *m*/*z* range 400–10,000 with a transient domain of 1 mega-word data points and estimated resolving power of 270,000 (at *m*/*z* 400). MALDI-ISD spectra were phased corrected and processed using DataAnalysis and Biotools software. Fragment ions were identified with mass tolerance 50 ppm and sequence coverage of the fusion protein is shown in Fig. 6.

### 4.3 ETD MS/MS analysis of IdeS-digested protein

The fusion protein sample was diluted to 1 µg/µL in 50 mM ammonium bicarbonate solution and digested with enzyme IdeS at ratio of 1 enzyme unit to 1 µg protein for 2 h at 37 °C. 20 mM DDT was added to cleave disulfide bonds at 80 °C for 15 min. The sample was further concentrated using 10 kDa MWCO filter and subject to detergent removal. The sample buffer was exchanged to 5% ACN/0.1% FA and diluted to final concentration of approximately 7 µg/µL in 50%ACN/0.1% FA. IdeS digestion and DTT reduction cut the fusion protein to half the size producing N-terminal and C-terminal fragments.

Accurate mass measurement of the IdeS/DTT treated sample was performed by ESI FT-ICR. Mass spectra were collected in magnitude mode with a transient domain of one mega-word data points to cover *m*/*z* range 400–4000. Seven glycoforms were identified from the C-terminus at multiple charge states. Table 1 represents glycosylated protein species detected from 17+ charge peaks and their glycan nomenclature and structures are shown in Fig. 5.

**Fig. 7:**
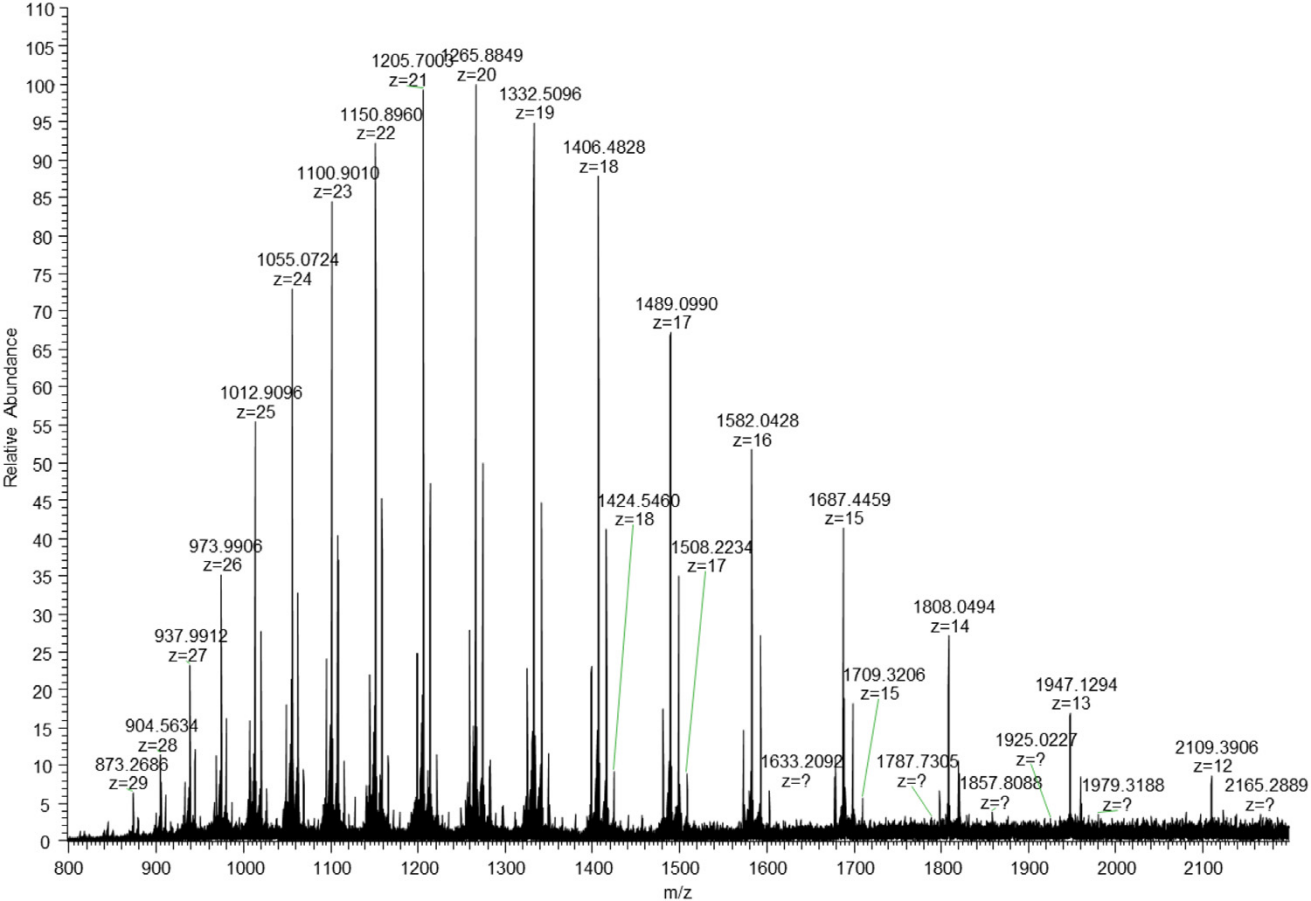
LC-MS mass spectrum of IdeS/DDT treated fusion protein. Charge state distribution of glycosylated protein species from of C-terminal fragment is shown. The mass spectrum was produced from accumulation of 20 scans over an *m*/*z* range 600–3500 at a resolving power of 120,000.

**Fig. 8:**
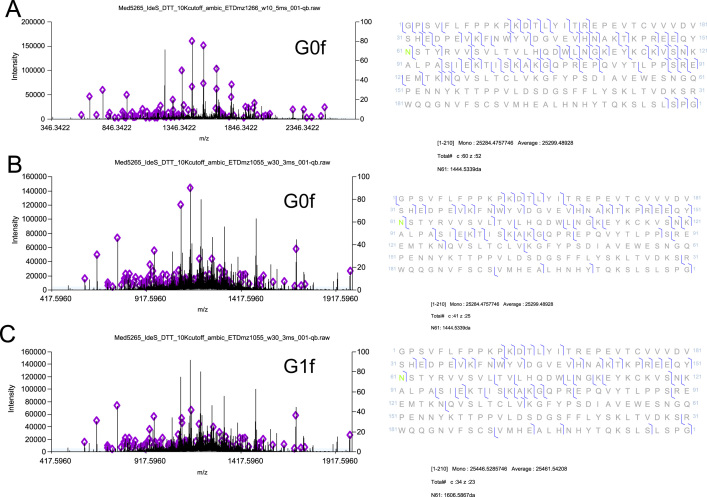
LC-MS/MS ETD mass spectra of (A) precursor *m*/*z* 1266, and (B and C) *m*/*z* 1055 were searched against C-terminal fragment of DTT/IdeS treated fusion protein. Identified c- and z- fragment ion series are indicated in diamonds in the mass spectra and relevant sequence identifications are shown in the associated sequences to the right of each mass spectrum. Detection of glycosylated protein species, G0f and G1f was confirmed on N207 (in green) with C-terminal lysine truncation.

The sample was also directly infused into the Orbitrap Elite. Data were collected in positive ion mode for 350–3500 *m*/*z* range at resolving power of 120,000 (Fig. 7). Precursor masses of the glycoforms were isolated for ETD fragmentation. ETD mass spectra were acquired with 5 microscans at resolving power of 120,000 and processed by MASH Suite for sequence coverage analysis. Manual interpretation of the ETD spectra confirmed that glycosylation by G0f or G1f occurred on a site N207 and the protein has a C-terminal lysine truncation (Fig. 8).

## Figures and Tables

**Table 1:** Glycosylated protein species detected by Fourier transform ion cyclotron resonance (FT-ICR) accurate mass measurement of DTT/IdeS treated fusion protein from the 17+ charge state. Highlighted in bold are the most abundance glycoforms detected on the C-terminal fragment of the protein. Additional information regarding the glycan nomenclature and structure are provided in the Fig. 6.

Potential glycosylated protein species	Observed *m*/*z*	Charge (z)	Observed monoisotopic mass [M+H]^+^	Theoretical monoisotopic mass [M+H]^+^	Mass error between observed and theoretical	
(Da)	(ppm)
**G0**	1479.64124	17+	25137.77353	25139.397044	−1.62	−64.58
**G0f**	1488.28997	17+	25284.83767	25282.454953	2.38	94.24
**G1f**	1497.76750	17+	25445.88614	25444.507776	1.38	54.17
**G2f**	1507.29477	17+	25607.90766	25606.560599	1.35	52.61
G1f+NANA	1514.88175	17+	25736.94787	25735.063193	1.88	73.23
G2f+NANA	1524.41738	17+	25899.02107	25897.656016	1.37	52.71
G2f+NANA2	1541.54188	17+	26190.11349	26188.751433	1.36	52.01
